# Quantitative analysis of the major constituents in Chinese medicinal preparation SuoQuan formulae by ultra fast high performance liquid chromatography/quadrupole tandem mass spectrometry

**DOI:** 10.1186/1752-153X-7-131

**Published:** 2013-07-30

**Authors:** Feng Chen, Hai-long Li, Yong-Hui Li, Yin-Feng Tan, Jun-Qing Zhang

**Affiliations:** 1 School of Pharmacy, Hainan Medical University, Hainan Provincial Key Laboratory of R&D of Tropical Herbs, Haikou 571101, China

**Keywords:** Fructus *Alpiniae Oxyphyllae*, *Radix Linderae*, SuoQuan formulae, UFLC-MS/MS, Alkaloids, Diarylheptanoids, Flavonoids, Lactones, Naphthalenone

## Abstract

**Background:**

The SuoQuan formulae containing Fructus *Alpiniae Oxyphyllae* has been used to combat the urinary incontinence symptoms including frequency, urgency and nocturia for hundreds of years in China. However, the chemical information was not well characterized. The quality control marker constituent only focused on one single compound in the current Chinese Pharmacopeia. Hence it is prudent to identify and quantify the main constituents in this herbal product. This study aimed to analyze the main constituents using ultra-fast performance liquid chromatography coupled to tandem mass spectrometry (UFLC-MS/MS).

**Results:**

Fourteen phytochemicals originated from five chemical classes constituents were identified by comparing the molecular mass, fragmentation pattern and retention time with those of the reference standards. A newly developed UFLC-MS/MS was validated demonstrating that the new assay was valid, reproducible and reliable. This method was successfully applied to simultaneously quantify the fourteen phytochemicals. Notably, the content of these constituents showed significant differences in three pharmaceutical preparations. The major constituent originated from each of chemical class was isolinderalactone, norisoboldine, nootkatone, yakuchinone A and apigenin-4’,7-dimethylther, respectively. The variation among these compounds was more than 1000 times. Furthermore, the significant content variation between the two different Suoquan pills was also observed.

**Conclusion:**

The proposed method is sensitive and reliable; hence it can be used to analyze a variety of SuoQuan formulae products produced by different pharmaceutical manufacturers.

## Background

Urinary incontinence is common and costly. More than 200 million people worldwide live with this disorder, causing significant detrimental effects on their quality of life [1,2]. Involuntary urine loss has been reported to occur in 30.9% of women and in 3%–10% of men in the mainland of China [3]. Whilst a conservative approach to the treatment of incontinence is justified in almost all cases drug therapy such as antimuscarinic agents remains integral in patients complaining of overactive bladder or bothersome stress urinary incontinence [4]. On the other hand, some traditional Chinese herbs, such as fruits of *Alpinia oxyphylla* Miq. (Zingiberaceae, known as Yizhi in Chinese), are recommended for improving frequent urination and/or enuresis symptoms in current Chinese pharmacopoeia [5]. *A. oxyphylla* Fructus is usually used in compound formulae. Among them, the most famous is SuoQuan pills, which was first described in Chinese canonical medicine about 800 years ago for the treatment of different urinary incontinence symptoms including frequency, urgency and nocturia. This formulae consists of three herbs: *A. oxyphyllae* Fructus, *Radix linderae* (Lauraceae) and *Dioscorea opposite* (Dioscoreaceae) and as one of essential herbal drugs has been documented in Chinese pharmacopeia [6]. In China, the State Food and Drug Administration (SFDA) has approved nine pharmaceutical manufacturers to produce this Chinese patent medicine in the dosage forms as pills and capsules (http://app1.sfda.gov.cn/datasearch/face3/base.jsp). However, the chemical information in these products is not well characterized. Identification of main constituents is helpful in optimizing manufacturing procedures, ensuring batch consistency and fostering understanding of the clinical effects of the herbal product.

According to the principle of *jun-chen-zuo-shi*, the most influential theory of traditional Chinese medicine, the *A. Oxyphyllae* Fructus is used as the *jun* (emperor) herb and the *R. linderae* as the *chen* (minister) herb in the SuoQuan pills formulae [7]. Chemical analysis reveal that *A. oxyphyllae* Fructus contains flavonoids, diarylheptanoids, sesquiterpenes, volatile oil, steroids and their glycosides, *etc.*[8,9]. Isoquinoline alkaloids, sesquiterpene lactones, and flavonoids are the main bioactive components discovered in *R. linderae*[10,11]. Modern pharmacological studies have demonstrated that the kernels of *A. oxyphylla* possess anti-inflammatory function [12,13], and the diarylheptanoids (yakuchinone A and B) might be the active components [14-16]. Meanwhile, the alkaloids (*e.g.*, norisoboldine) [17-20] and lactones (*e.g.*, isolinderalactone) [21] from *R. linderae* can effectively alleviate inflammation. These constituents might have potential therapeutic roles for the prevention and treatment of incontinence. However, current chemistry quality control of SuoQuan pills focused only on quantitative analysis of linderane [6], and the results were inadequate for the whole quality assurance purposes. Therefore, a powerful analysis approach for identification and simultaneous determination of these multiple constituents occurred in SuoQuan formulae is urgently needed to ensure the quality control, as well as efficacy and safety, of the Chinese patent drug.

In our laboratory, we have systematically isolated the natural products of *A. oxyphylla* fruits [9] and characterized its nucleobases and nucleosides contents collected from different cultivation regions [8]. Pharmacological studies revealed that flavonol izalpinin could inhibit the muscarinic receptor-related detrusor contractile activity (published elsewhere). In the present work, a new UFLC-MS/MS method was developed and validated for simultaneous determination of 14 phytochemicals present in *A. Oxyphyllae* Fructus or *R. linderae*. Using the newly developed method, the content levels of 14 phytochemicals in SuoQuan pills were determined and compared. Notably, the content of these constituents demonstrated significant differences in three SuoQuan formulae products.

### Experimental

#### ***Chemicals and reagents***

Reference standards of linderane (purity, 98%; similarly hereinafter) and norisoboldine (96.9%) were obtained from the National Institutes for Food and Drug Control (Beijing, China). Nootkatone (98%) and boldine (99%) were purchased from Sigma-Aldrich (St Louis, MO, USA). Atractylenoide III (98%) and isolinderalactone (98%) were obtained from Tianjin Bestbiotech Co. Ltd. (Tianjin, China). Yakuchinone A (98%), Yakuchinone B (98%) and oxyphyllacinol (98%) were purchased from Chenfun Medical Technology (Shanghai) Co., Ltd. (Shanghai, China). Tectochrysin, izalpinin, chrysin, kaempferide and apigenin-4’,7-dimethylther were separated and identified from *A. Oxyphyllae* by Prof. Zhang (Hainan Provincial Key Laboratory of R&D on Tropical Medicinal Plants, Haikou, China) and the purity of these compounds were > 98%. HPLC-grade methanol and acetonitrile were products of Sigma-Aldrich (St Louis, MO, USA). HPLC-grade formic acid was purchased from Aladdin Industrial Inc. (Shanghai, China). HPLC-grade water was prepared by double-distillation of deionized water. The other chemical reagents of analytical grade or better were obtained from Hainan YiGao Instrument Co., Ltd (Haikou, China). The utilized SuoQuan pills (SQP) and SuoQuan capsules (SQC) are commercially available *A. Oxyphyllae* Fructus products. SQP-1 were obtained from Guangdong YiHeTang Pharmaceutical Co., Ltd. (lot no.20111210, expiration: 2013/11; Chinese SFDA ratification no.Z44023146; Zhongshan, Guangdong Province, China) and SQP-2 were purchased from Guangdong HuaTianBao Pharmaceutical Group (lot no.1110103, expiration: 2014/09; Chinese SFDA ratification no. Z44023538; Foshan, Guangdong Province, China). Twenty pills weigh 1 g for both SQP-1 and SQP-2. The SQC were purchased from Hansen Pharm. (lot no.110602, expiration: 2013/05; Chinese SFDA ratification no.Z19991039; Yiyang, Hunan Province, China) and each capsule contains 0.3 g of solid.

#### ***Construction of standard curves***

Blank solvent (acetonitrile or methanol) was spiked with the 14 test compounds to generate a nominal concentration of 1 mg/mL for each compound. Then, each stock solution was diluted step by step with methanol to prepare a sequence of standard solutions. All solutions were stored at -4°C before analysis. Standard curves were constructed using weighted (1/*X* or 1/*X*^*2*^) linear regression of the peak areas of the phytochemical analyte (*Y*) against the corresponding nominal concentration of the analyte (*X*, ng/mL).

#### ***Sample preparation***

Samples of 0.2 g (accurately weighed) of dry and powdered SQP or SQC were macerated with 10 mL of methanol and then ultrasonicated twice or triple for 30 min every time. Between each ultrasonication, the residue was filtered and washed with 1 mL of methanol. The resulting extract solution were combined and then centrifuged. The extract solution was diluted with methanol from 10 to 100 times before analysis.

#### ***UFLC-MS/MS***

An AB-SCIEX API 4000^+^ mass spectrometer (AB-Sciex, Toronto, Canada) interfaced via a Turbo V ion source with a Shimadzu Prominence UFLC chromatographic system (Shimadzu Corporation, Kyoto, Japan), which is equipped with two LC-20AD pumps, a model DGU-20A_3R_ degasser unit, a SIL-20A HT autosampler and a CTO-20A column oven. The AB-SCIEX Analyst software packages were used to control the UFLC-MS/MS system, as well as for data acquisition and processing. The mass was commonly calibrated every one month using polytyrosine glycol as standard in our laboratory.

**Figure 1:**
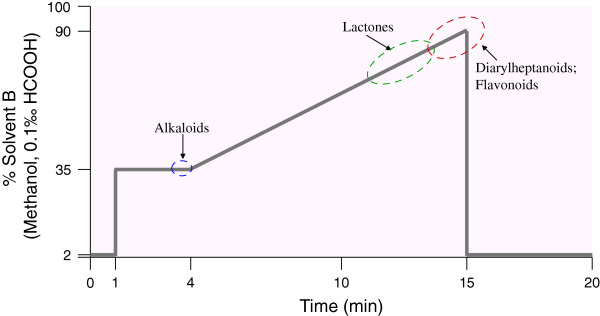
**Solvent B (%) concentration-time profile of a 20-min LC binary gradient program.** Solvent B containing 0.1‰ formic acid and the gradient program was as fallows: from 0% B to 2% B in 0.01 min, hold for 1 min; from 2% B to 35% B in 0.01 min, hold for 3min; from 35% B to 90% B in 11 min; back to 2% B in 0.01 min; maintain 4.99 min. The effluent was delivered at 0.3 mL/min throughout the gradient program and the column was maintained at 40°C. The dashed ovals showed the eluted analytes of different types of compounds (*i.e.*, alkaloids, lactones, flavonoids and diarylheptanoids) under this optimized UFLC-MS/MS conditions.

Chromatographic separations of prepared samples were achieved using a Shim-pack XR-ODS column (2.0 mm i.d × 100 mm) maintained at 40°C. The LC mobile phases included H_2_O containing 0.1‰ formic acid for solvent A, and methanol containing 0.1‰ formic acid for solvent B. As shown in Figure 1, a specially designed LC binary gradient program was used to separate the phytochemicals and the effluent was delivered at 0.3 mL/min throughout the gradient program.

**Figure 2:**
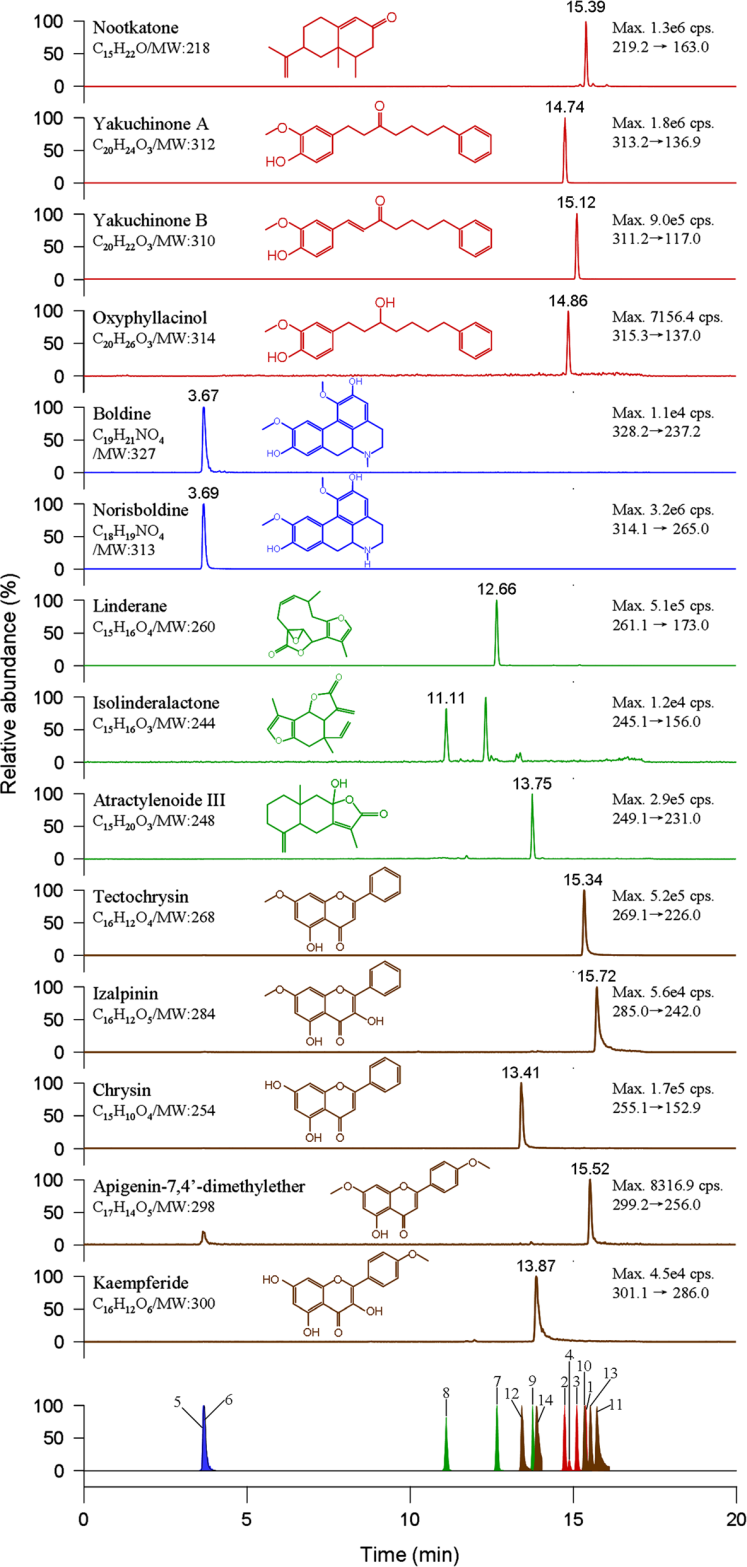
**Typical UFLC-MS/MS chromatograms of 14 phytochemicals from a SQC sample.** They were identified by the comparison of the retention times and mass spectra (MRM mode) with the corresponding pure compounds. Peak from 1 to 14 in the bottom panel donates nootkatone, yakuchinone A, yakuchinone B, oxyphyllacinol, boldine, norisoboldine, linderane, isolinderalactone, atractylenoide III, tectochrysin, izalpinin, chrysin, apigenin-4’,7-dimethylther and kaempferide, respectively.

The mass spectrometer was operated in the positive ion ESI mode with multiple reaction monitoring (MRM) for all the analytes. The pneumatically nebulized ESI spraying was achieved by using inner coaxial nebulizer N_2_ gas of 55 psi through a TurboIonSpray probe, a high voltage of + 5.5 kV applied to the sprayer tip, and heated dry N_2_ gas of 55 psi at 550°C from two turbo heaters adjacent to the probe. To prevent solvent droplets from entering and contaminating the ion optics, a curtain N_2_ gas of 25 psi was applied between the curtain plate and the orifice. The collision gas flow was set at level 4. The precursor-to-product ion pairs (Figure 2) used for MRM of nootkatone, yakuchinone A and B, oxyphyllacinol, tectochrysin, izalpinin, chrysin, kaempferide, apigenin-4’,7-dimethylther, boldine, norisoboldine, linderane, isolinderalactone and atractylenoide III were *m/z* 219.2→163.0 (the optimal collision energy, 22 V), 313.2→136.9 (13 V), 311.2→117.0 (30 V), 315.3→137.0 (22 V), 269.1→226.0 (43.5 V), 285.0→242.0 (43 V), 255.1→152.9 (42 V), 301.1→286.0 (37 V), 299.2→256.0 (45 V), 328.2→237.2 (20 V), 314.1→265.0 (25 V), 261.1→173.0 (18 V), 245.1→156.0 (29 V) and 249.1→231.0 (28 V) respectively, with a scan time of 20 ms for each ion pair.

## Results and discussion

### Selection of the extraction method

Studies designed to investigate the optimal method for extraction have been published previously [8,11,22]. In this study, therefore, we chose the methanol as the solvent and the ultrasonic extraction (40 KHz, 80 W) was used as the extraction method. The number of extraction and extraction time were evaluated. The results showed that the recoveries of the 14 phytochemicals were about 90% by single extraction but almost 100% through twice extraction for SQC samples. Meanwhile, the investigated constituents were extracted completely through triple extraction for SQP sample.

### Selection of UFLC-MS/MS condition

The parent ion *m/z* of each analyzed compound was selected by direct injection based on the optimization of MS/MS parameters. We found that 14 phytochemicals in methanol or acetonitrile could form [M+H]^+^ quasi-molecular ions with relative high abundance under the ESI^+^ mode. Based on the conformation of parent ions, the optimization of product ions was performed in the MS2 scan mode. Finally, the precursor-to-product ion pairs were optimized in the MRM scan mode.

Shim-pack XR-ODS column (2.0 mm i.d × 100 mm) was used to separate the target analytes. The column oven was maintained at 40°C in order to achieve good separation. The LC mobile phases containing 0.1‰ formic acid was helpful to improve the peak tailing of flavonoids. The UFLC-MS/MS chromatograms of 14 phytochemicals from a SQC sample were shown in Figure 2. They were identified by the comparison of the retention time and mass spectra with the corresponding pure compounds.

## Method validation

### Calibration curves, limits of detection and quantification

Every calibration curve was performed with six different concentrations in quintuplicate and the calibration graphs were plotted after linear regression of the peak areas versus the corresponding concentration of each analyte. Calibration curves were linear with correlation coefficient (*R*^2^) >0.99 for all analytes. Table 1 shows the characteristic parameters for linear dynamic ranges and coefficient. The limit of detection (LOD) and limit of quantification (LOQ) was the concentration of an analyte at which its signal-to-noise ratio were detected as 3:1 and 10:1, respectively. They were achieved by serial dilution of sample solution using the described UFLC-MS/MS conditions.

**Table 1:** Characteristic parameters of the investigated compounds analyzed with the UFLC-MS/MS system

**Analytes**	**Linear ranges (ng/mL)**	**Linear equation (weight, 1/*X *or 1/*X***^***2***^**)**	***R***^**2**^	**LOD (ng/mL)**^**a**^	**LOQ (ng/mL)**^**b**^
Nootkatone	2.0–1000	*Y*=3980*X*+13000	0.995	0.40	2.00
Yakuchinone A	2.0–2000	*Y*=7070*X*+10500	0.986	0.20	2.00
Yakuchinone B	2.0–1000	*Y*=7080*X*+5.450	0.993	0.27	2.00
Oxyphyllacinol	5.83–2330	*Y*=23.4*X*+800	0.998	5.83	11.7
Boldine	5.0–2000	*Y*=72.80*X*+59.40	0.996	2.00	5.00
Norisoboldine	1.94–1940	*Y*=6940*X*+19300	0.993	0.19	1.94
Linderane	2.16–2160	*Y*=3590*X*+630.0	0.995	0.43	2.16
Isolinderalactone	2.6–1040^c^	*Y*=0.604*X*+0.785	0.995	1.04^c^	2.60^c^
Atractylenoide III	2.0–2000	*Y*=1630*X*+1440	0.997	2.00	5.00
Tectochrysin	2.07–2070	*Y*=6470*X*+1190	0.998	0. 21	2.07
Izalpinin	2.0–2000	*Y*=1490*X*-2460	0.998	1.00	2.00
Chrysin	2.0–2000	*Y*=1560*X*+106.0	0.996	1.00	2.00
Apigenin-4’,7-dimethylther	5.0–2000	*Y*=76.00*X*-134.0	0.994	2.00	5.00
Kaempferide	5.0–2000	*Y*=455.0*X*-710.0	0.999	2.00	5.00

Note: ^a^Limits of detection; ^b^Limits of quantification; ^c^Concentration unit: μg/mL.

### Precision, repeatability and stability

The precisions of the developed method were evaluated by analyzing the SQC solution in six replicates during a single day and by duplicating the experiments on three consecutive days. As shown in Table 2, the intra- and inter-day precision of the analytical method were quite good for all the investigated constituents, i.e., 0.26–5.54% and 2.06–5.32%, respectively. The repeatability was conducted by calculating the concentrations of six freshly prepared SQC samples. As a result, a good repeatability was achieved for the new method with RSD no more than 10% (Table 3). The stability of the analytes was evaluated under conditions mimicking situations likely to be encountered during sample storage and the analytical process. In this study, the freshly prepared SQC sample was stored at ambient temperature and injected into UFLC-MS/MS system at intervals 0, 6, 12, 24 and 48 h. The results (RSD <5%, Table 3) showed the sample solution was quite stable within two days.

**Table 2:** Intra-day and inter-day precision of the investigated compounds’ in SQC sample

**Analytes**	**Day 1 (n=6)**		**Day 2 (n=6)**		**Day 3 (n=6)**		**Inter-day (n=18)**	
	**Mean ± SD (ng/mL)**	**RSD (%)**	**Mean ± SD (ng/mL)**	**RSD (%)**	**Mean ± SD (ng/mL)**	**RSD (%)**	**Mean ± SD (ng/mL)**	**RSD (%)**
Nootkatone	79.7 ± 2.0	2.51	75.9 ±1.0	1.32	75.0 ± 0.6	0.80	77.2±2.6	3.37
Yakuchinone A	820 ± 32	3.90	833 ± 23	2.76	786 ± 36	4.58	815 ± 34	4.17
Yakuchinone B	36.5 ± 0.9	2.47	36.8 ± 1.5	4.08	33.0 ± 1.3	3.94	35.7 ± 1.9	5.32
Oxyphyllacinol	613 ± 28	4.57	625± 12	1.92	620 ± 25	4.03	619 ± 22	3.55
Boldine	346 ± 12	3.47	338 ± 13	3.85	325 ± 18	5.54	338 ± 16	4.73
Norisoboldine	280 ± 10	3.57	279 ± 9	3.23	262 ± 8	3.05	270 ± 12	4.44
Linderane	20.8 ± 0.4	1.92	20.4 ± 0.7	3.43	19.1 ± 0.8	4.19	20.2 ± 0.9	4.46
Isolinderalactone^a^	121 ± 6	4.96	115 ± 4	3.48	114 ± 1	0.88	117 ± 5	4.27
Atractylenoide III	63.3 ± 2.2	3.48	61.2 ± 1.3	2.12	63.4 ± 2.8	4.42	62.6 ± 2.3	3.67
Tectochrysin	466 ± 17	3.65	465 ± 9	1.94	430 ± 20	4.65	456 ± 22	4.82
Izalpinin	78.3 ± 0.9	1.15	78.8 ± 1.2	1.52	75.9 ± 0.2	0.26	77.8 ± 1.6	2.06
Chrysin	29.9 ± 0.9	3.01	30.7 ± 1.5	4.89	29.3 ± 0.4	1.37	30.0 ± 1.1	3.67
Apigenin-4’,7-dimethylther	672 ± 28	4.17	653 ± 17	2.60	610 ± 9	1.48	649 ± 33	5.08
Kaempferide	53.5 ± 1.8	3.36	53.5 ± 0.7	1.31	52.6 ± 1.6	3.04	53.3 ± 1.4	2.63

Note: ^a^Concentration unit: μg/mL.

**Table 3:** Stability and repeatability of the investigated compounds in SQC sample

**Analytes**	**Stability (n=6)**		**Repeatability (n=6)**	
	**Mean ± SD (ng/mL)**	**RSD (%)**	**Mean ± SD (ng/mL)**	**RSD (%)**
Nootkatone	77.3 ± 2.8	3.62	71.2 ± 2.7	3.79
Yakuchinone A	818 ± 25	3.06	811 ± 42	5.18
Yakuchinone B	36.3 ± 1.7	4.68	36.4 ± 1.8	4.95
Oxyphyllacinol	1015 ± 44	4.33	637 ± 3	0.47
Boldine	342 ± 13	3.80	315 ± 10	3.17
Norisoboldine	271 ± 13	4.80	194 ± 17	8.76
Linderane	20.6 ± 0.58	2.82	190 ± 6	3.16
Isolinderalactone^a^	116 ± 4	3.45	105 ± 3	2.86
Atractylenoide III	63.7 ± 1.6	2.51	86.7 ± 3.1	3.58
Tectochrysin	457 ± 20	4.38	400 ± 15	3.75
Izalpinin	78.1 ± 1.6	2.05	85.1 ± 1.5	1.76
Chrysin	29.8 ± 1.3	4.36	256 ± 13	5.08
Apigenin-4’,7-dimethylther	656 ± 32	4.88	576 ± 27	4.69
Kaempferide	53.3 ± 1.6	3.00	62.4 ± 1.1	1.76

Note: ^a^Concentration unit: μg/mL.

### Recovery

The recovery of known amounts of standards added to samples was used to assess the new method accuracy. Six portions of SQC or SQP sample were spiked with the mixed standards of 14 phytochemicals. Then the samples were pretreated as described in **“**Sample preparation**”** section, and the results are summarized in Table 4. All the recoveries were in the range of 98.2‒105% for SQC sample, 95.6‒102% for SQP sample, with the RSDs 0.80‒4.93% and 1.00‒4.76%, respectively.

**Table 4:** Recoveries of the investigated compounds in SQC or SQP samples

**Analytes**	**SQC sample (n=6)**				**SQP sample (n=6)**			
	**Measured (μg) (Mean ± SD)**	**Added (μg)**	**Recovery (%, mean)**	**RSD (%)**	**Measured (μg) (Mean ± SD)**	**Added (μg) (Mean ± SD)**	**Recovery (%, mean)**	**RSD (%)**
Nootkatone	5.00 ± 0.13	5.00	100	2.67	5.06 ± 0.19	5.00	101	3.84
Yakuchinone A	4.91 ± 0.14	5.00	98.2	2.80	4.77 ± 0.15	5.00	95.3	3.23
Yakuchinone B	4.97 ± 0.13	5.00	99.4	2.52	5.01 ± 0.24	5.00	100	4.76
Oxyphyllacinol	5.93 ± 0.22	5.83	102	3.64	5.71 ± 0.24	5.83	98.0	4.21
Boldine	5.00 ± 0.11	5.00	100.	2.13	5.04 ± 0.09	5.00	101	1.85
Norisoboldine	4.91 ± 0.24	4.85	101	4.93	4.81 ± 0.18	4.85	99.2	3.70
Linderane	5.37 ± 0.19	5.40	99.4	3.60	5.41 ± 0.21	5.40	100	3.84
Isolinderalactone	73.9 ± 2.2	75.0	98.5	3.02	74.8 ± 2.4	75.0	99.7	3.23
Atractylenoide III	5.23 ± 0.20	5.00	105	3.81	5.05 ± 0.15	5.00	101	3.04
Tectochrysin	3.96 ± 0.17	4.00	98.9	4.38	3.82 ± 0.08	4.00	95.6	2.18
Izalpinin	2.53 ± 0.07	2.50	101	2.77	2.51 ± 0.06	2.50	100	2.23
Chrysin	5.11 ± 0.13	5.00	102	2.45	5.13 ± 0.05	5.00	102	1.00
Apigenin-4’,7-dimethylther	5.11 ± 0.15	5.00	102	2.89	5.04 ± 0.17	5.00	101	3.40
Kaempferide	7.37 ± 0.06	7.05	104	0.80	7.06 ± 0.21	7.05	100	2.92

### Quantification of 14 phytochemicals from three SuoQuan formulae preparations

**Figure 3:**
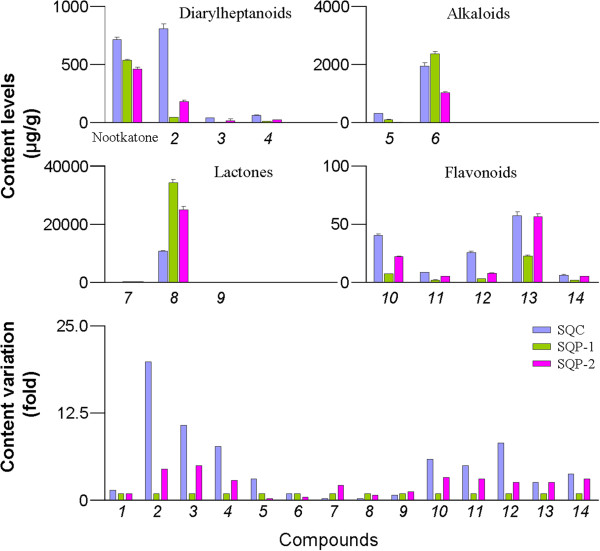
**The content variation of 14 phytochemicals originated from SQC, SQP-1 and SQP-2 samples.** In the bottom panel, the content level variation was normalized based on the contents in SQP-1 sample.

The newly validated method was applied to simultaneously determine the 14 compounds in three commercial SuoQuan formulae products from different company. The results are summarized in Table 5 and Figure 3. Our results revealed that the contents of the 14 compounds are significantly different. The overall content level was as follows: lactones > alkaloids > naphthalenone (nootkatone) > diarylheptanoids > flavonoids, albeit the dosage forms (*i.e.*, capsules or pills). Accordingly, the highest content constituent of each type of compound was isolinderalactone, norisoboldine, nootkatone, yakuchinone A and apigenin-4’,7-dimethylther, respectively. Moreover, the content variation was more than 1500 times among these phytochemicals .

**Table 5:** Contents of 14 phytochemicals from three SuoQuan formulae products

**Analytes**	**Content (μg/g)**		
	**SQC**	**SQP-1**	**SQP-2**
Nootkatone	712 ± 27	535 ± 12	461 ± 18
Yakuchinone A	811 ± 42	40.7 ± 2.0	181 ± 8.1
Yakuchinone B	36.4 ± 1.8	3.42 ± 0.09	16.5 ± 0.3
Oxyphyllacinol	63.7 ± 3.0	8.19 ± 0.38	23.8 ± 1.0
Boldine	315 ± 10	102 ± 5	18.4 ± 0.5
Norisoboldine	1940 ± 116	2360 ± 98	1036 ± 40
Linderane	19.0 ± 0.6	132 ± 1	266 ± 10
Isolinderalactone	10517 ± 264	34225 ± 1096	24875 ± 1228
Atractylenoide III	8.67 ± 0.31	12.2 ± 0.6	13.8 ± 0.6
Tectochrysin	40.0 ± 1.5	6.93 ± 0.29	21.9 ± 0.4
Izalpinin	8.51 ± 0.15	1.74 ± 0.07	5.13 ± 0.10
Chrysin	25.6 ± 1.3	3.15 ± 0.13	7.79 ± 0.40
Apigenin-4’,7-dimethylther	57.6 ± 2.73	22.4 ± 1.0	56.2 ± 2.8
Kaempferide	6.24 ± 0.11	1.67 ± 0.09	4.97 ± 0.21

On the other hand, the manufacturing process influenced the content levels. Besides for lactones, the other types of compound had higher concentration in SQC samples than SQP samples. SQP is a Chinese traditional medical mixture containing the powder of *A. oxyphyllae* Fructus, *R. linderae* and *D. opposite* and the manufacturing process dose not involve the extraction technology. Therefore, all the constituents derived from three herbal powders are transformed into the final pills. On the contrary, the SQC is manufactured through refluxing extraction method with ethanol and the constituents that can dissolved in ethanol are differentially transformed into the final capsules according to the theory of “like dissolves like”. As a result, most of the investigated constituents were concentrated and the content variation in SQC sample might smaller than the SQP sample (Figure 3).

As shown in Figure 3, it was worth noting that the significant content variation between the SQP-1 sample and SQP-2 sample was observed. As for linderane, a marker compound for quality control in current Chinese Pharmacopeia [6], the mean content levels (132 μg/g for SQP-1 and 266 μg/g for SQP-2) were superior to the mandatory criteria (*i.e.*, no less than 90 μg/g) albeit the 2-fold difference between the two products. However, for the most of the other constituents, the content levels in SQP-2 sample were larger than SQP-1 sample. The diarylheptanoids’ content levels in SQP-2 were 3–5 times higher than SQP-1. Similarly, this data for flavonoids was 2–3 times. The possible explanation for these discrepancies could be that the herbal raw materials were different between the two companies. Thus, strict quality control for raw material and adequate in-process controls should be properly carried out. In the cases where the active principles are unknown, marker substance(s) should be established for analytical purposes.

In the present work, we also found other compounds occurred in the SQC and SQP sample including linderagalactone D, linderagalactone C, hydroxylindestenolide, neolinderalactone, reticuline and norboldine. These phytochemicals were well separated and their precursor-to-product ion pairs were set at 263.5→227.2, 263.5→217.1, 247.7→229.2, 245.5→199.4, 330.2→192.2 and 314.0→265.3 according to the data reported in literature [11,22]. However, these compounds were not quantified because the corresponding reference standards were not available.

## Conclusion

In summary, a new UFLC-MS/MS method was developed and validated. The proposed method was sensitive and reliable and then successfully applied to simultaneously quantify the 14 phytochemicals occurred in SuoQuan formulae. Significant content variations were observed. Manufacturing processes influenced the content levels between the different dosage forms. The quality of raw materials play a pivotal role in guaranteeing the consistently good quality of herbal preparations. Sufficient chemical information and assay for particular constituents or markers are helpful in understanding of the clinical effects of the herbal product.

## Abbreviations

UFLC-MS/MS: Ultra fast liquid chromatography-tandem mass spectrometry; RSD: Relative standard variation; SQC: SuoQuan capsules; SQP: SuoQuan pills.

## Competing interests

There are no competing interests to declare.

## Authors’ contributions

CF and LHL were the primary contributors to this manuscript. CF and LHL were responsible for preparing the first draft of the manuscript and performed most of the experimentation and analysis while also being involved heavily in data acquisition and interpretation. ZJQ was involved in design of the experiments and provided critical advice on operation of the analytical equipment due to previous expertise. LYH and TYF had a significant role in development of the experiments and interpretation of results. All authors read and approved the final manuscript.
